# Clinical results of reverse shoulder arthroplasty after failed arthroscopic rotator cuff repair compared to primary cases: a case–control study

**DOI:** 10.1186/s42836-025-00323-0

**Published:** 2025-08-01

**Authors:** Noriaki Shimada, Jun’ichi Inoue, Ryota Takei, Kazuo Saita, Hiroshi Inui

**Affiliations:** 1 Department of Orthopaedic Surgery, Saitama Medical Center, Saitama Medical University, Saitama, 350-8550 Japan; 2 Department of Orthopaedic Surgery, Kamifukuoka General Hospital, Saitama, 356-0011 Japan

**Keywords:** Reverse shoulder arthroplasty, Arthroscopic rotator cuff repair, Postoperative retear

## Abstract

**Background:**

Although reverse shoulder arthroplasty (RSA) is a popular treatment, its efficacy in patients with failed rotator cuff repair (ARCR) remains unclear. In this study, we aimed to evaluate the clinical results of RSA for following failed ARCR. We hypothesized that RSA after failed ARCR would lead to improved clinical outcomes comparable to those of RSA performed without prior surgeries.

**Methods:**

Between January 2017 and December 2022, 143 patients underwent RSA at our institution. We included 85 patients who met the study criteria and followed them for a minimum of 2 years. The patients were divided into two groups: those who underwent RSA for failed ARCR (group A: 25 patients; mean age, 77.7 years) and those who underwent primary RSA (group B: 60 patients; mean age, 77.9 years). The University of California, Los Angeles (UCLA) scores, Japanese Orthopaedic Association (JOA) scores, range of motion (ROM), Numerical Rating Scale (NRS) scores, and complication rates were compared between the two groups.

**Results:**

At the 2-year postoperative follow-up, both groups showed significant improvements in all items. Postoperative outcome or complication rate demonstrated no significant difference between group A and group B: UCLA scores (29.7 ± 3.9 vs 29.3 ± 3.6), JOA scores (87.4 ± 6.1 vs 87.4 ± 8.6), ROM forward elevation (129.1 ± 20.1 vs 133.9 ± 24.1), ROM external rotation (29.1 ± 12.7 vs 29.4 ± 10.7), ROM internal rotation (2.4 ± 1.0 points vs 2.3 ± 1.1 point), NRS scores (0.9 ± 1.2 vs 1.1 ± 1.3), and complication rates (4.0% vs 3.3%). Group A exhibited improvement in all items, and the results were comparable to those in group B.

**Conclusions:**

RSA in patients with prior rotator cuff repair demonstrated similar functional outcomes and complication rates to those in patients who underwent RSA without prior surgeries. The study demonstrated that prior ARCR would not be a negative predictor. For patients who are afraid of or cannot consent to artificial joint surgery, recommending ARCR first may be an option.

**Graphical Abstract:**

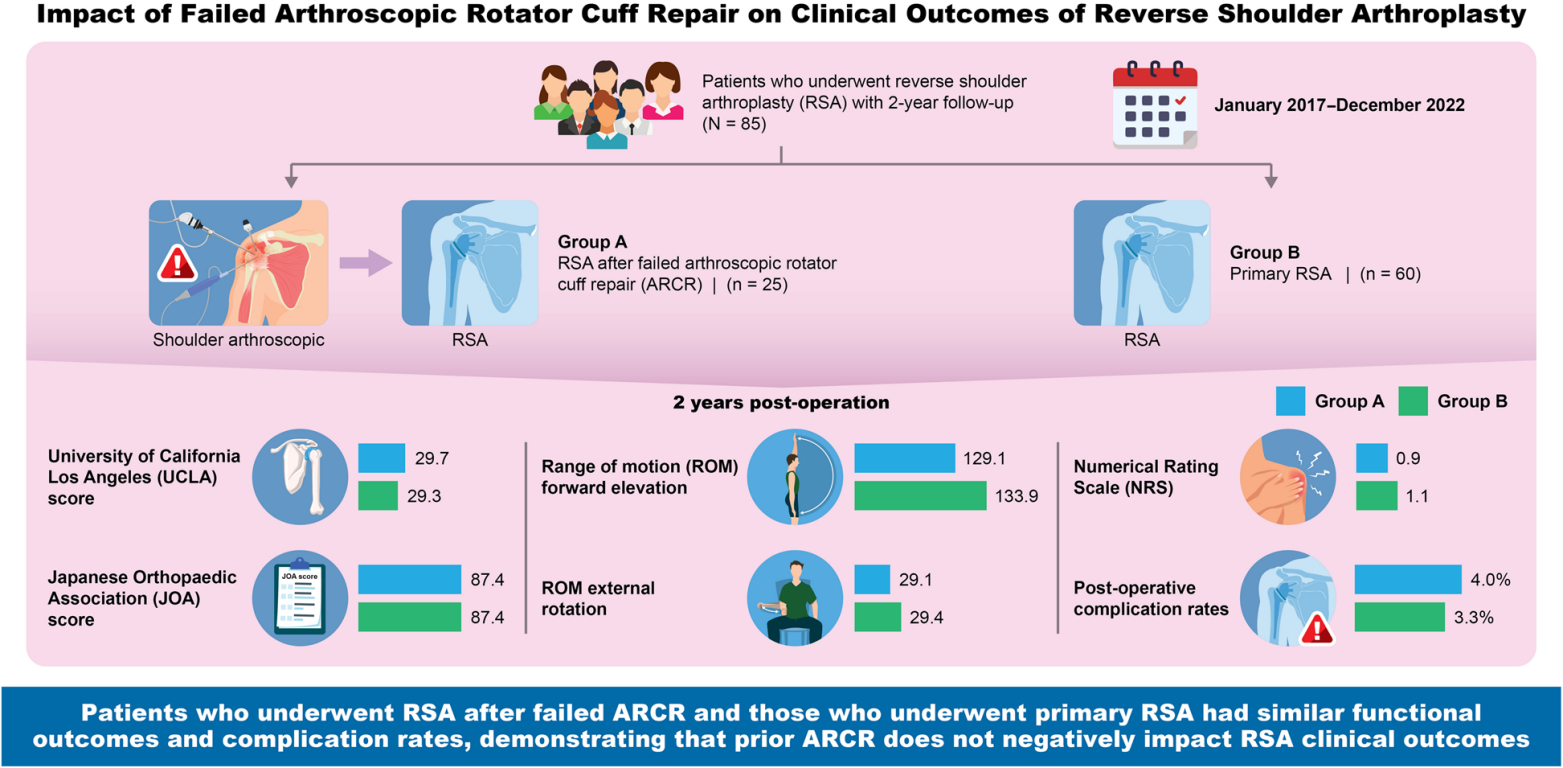

## Background

Reverse shoulder arthroplasty (RSA), introduced in the 1980 s, has demonstrated reliable outcomes, particularly for patients with cuff tear arthropathy (CTA). Indications for RSA have since expanded to include injuries such as massive rotator cuff tears, proximal humeral fractures, and fracture sequelae, with many studies reporting satisfactory results.

In Japan, RSA was introduced in 2014 and has rapidly gained popularity [1]. Notably, many studies have reported on the efficacy of RSA in Japan [1–3]. At our institution, RSA has been performed since 2015 and has consistently had positive effects and shown improvements in patient outcomes. Importantly, the number of RSA procedures performed at our institution has increased significantly, surpassing that of anatomical total shoulder arthroplasty or hemiarthroplasty.

Alternatively, arthroscopic rotator cuff repair (ARCR) has established itself as a minimally invasive standard treatment for rotator cuff tears and is considered the gold standard. However, re-injury after ARCR is not uncommon, and treatment strategies for postoperative retear remain controversial.

As RSA usage has increased, the number of RSA procedures performed after postoperative retear has also increased in our institution.

RSA is used as a salvage procedure in these cases. Some studies have assessed the impact of prior rotator cuff repair on RSA outcomes [4–11]. While options vary, there is no consensus on the effectiveness of RSA for such cases or the impact of prior ARCR. Some reports suggest no difference in outcomes [5, 8, 11, 12], while others indicate inferior outcomes for patients with prior rotator cuff repair [7, 9, 13]. Generally, revision surgeries are considered more challenging and may have inferior results compared with primary surgeries. Some studies have found RSA for failed arthroplasty or failed osteosyntheses to be inferior to primary surgeries [14–16]. However, the efficacy of RSA for patients with failed ARCR remains unclear. This study aimed to evaluate the outcomes of RSA after failed ARCR and to clarify the impact of prior ARCR on the effectiveness of RSA.

We hypothesized that RSA would significantly improve outcomes in patients with previous ARCR, including complication rates comparable to those of RSA without any prior shoulder surgeries.

## Methods

The current study was a retrospective observational study. The authors affirmed that this work followed The Code of Ethics of the World Medical Association (Declaration of Helsinki), and it was approved by the ethical committee of our institution. Written informed consent was obtained from all participants.

We defined failed ARCR as cases in which postoperative retear was confirmed by magnetic resonance imaging (MRI) and which interfered with daily life due to continuous pain or limitation of range of motion (ROM). All RSA was performed in both groups due to persistent pain, decreased active ROM, or inability to perform activities of daily living resulting from the combination of these conditions.

**Fig. 1:**
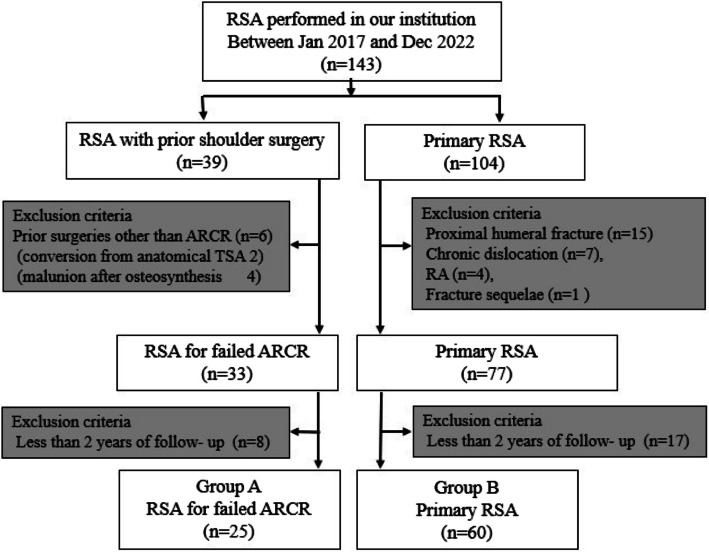
Flowchart of patients considered in this study

We included patients who underwent RSA between January 2017 and December 2022. During this period, 143 patients underwent RSA. Initially, the subjects were divided into two groups: patients with prior shoulder surgery and those undergoing primary RSA. Moreover, patients with a history of shoulder surgeries other than ARCR, cases of proximal humeral fracture, chronic dislocation, rheumatoid arthritis (RA), and fracture sequelae, were excluded. Furthermore, cases with less than 2 years of follow-up were excluded, resulting in 85 patients meeting the criteria. Finally, 25 patients with prior ARCR were included in group A, and 60 patients undergoing primary RSA were included in group B (Fig. 1). Specifically, we compared both groups. Group A comprised 11 men and 14 women, with a mean age of 77.7 ± 6.56 years. Group B comprised 22 men and 38 women, with a mean age of 77.9 ± 4.41 years. There were no significant differences in age, sex, or affected shoulder between the groups. In group A, the average interval between prior ARCR and RSA was 26.2 ± 18.0 months (Table 1).

**Table 1:** Patient demographic data

	Group A (*n* = 25)	Group B (*n* = 60)	*P*-value
Age, years	77.7 ± 6.56	77.9 ± 4.41	0.495
Sex (male/female)	11/14	22/38	0.724
Affected shoulder (right/left)	16/9	38/22	0.967
Average interval to RSA (Months)	26.2 ± 18	-	-

Group A: RSA for failed ARCR, Group B: Primary RSA

RSA procedures were performed using a standard deltopectoral approach with the same prosthesis (Aequalis Ascend Flex™, Stryker). Except in irreparable cases, the subscapularis tendon was repaired at the end of the procedure. ARCR procedures utilized the transosseous equivalent technique with suture anchors. In group A, rotator cuff retears were confirmed by MRI 6 months or 1 year postoperatively. Rotator cuff integrity was evaluated using Sugaya’s MRI-based classification system (Types I–V); we considered types IV and V as postoperative retear.

All surgeries were performed by a single surgeon (author S.N.) at a single institution (Saitama Medical Center, Saitama Medical University) under general anesthesia and an interscalene block, with patients positioned in the beach chair position.

Postoperatively, in all cases, the operative arm was supported in a sling with a small abduction pillow for 4 weeks. Passive ROM exercises began the day after surgery, and rehabilitation progressed based on postoperative pain with no limitations.

We compared the University of California, Los Angeles (UCLA) score, Japanese Orthopedic Association (JOA) score, ROM, numerical rating scale (NRS) scores, and complication rates between the groups. Additionally, we compared the UCLA score, JOA score, active ROM, and NRS scores preoperatively and at the 2-year postoperative follow-up. ROM measurements were obtained using standardized goniometric techniques with patients in the seated position, assessing forward elevation, external rotation, and internal rotation at 0° abduction. Specifically, for internal rotation, 10 points correspond to the eighth thoracic vertebra (T8), 9 points to T9, 3 points to the third lumbar vertebra (L3), and 0 points to the sacrum.

All statistical analyses were performed with EZR (Saitama Medical Center, Jichi Medical University, Saitama, Japan), which is a graphical user interface for R (The R Foundation for Statistical Computing, Vienna, Austria). More precisely, it is a modified version of R Commander designed to add statistical functions frequently used in biostatistics. According to the statistical power analysis, the estimated sample size was 24. Differences between the two groups were analyzed using the Mann–Whitney U test, chi-square test, and Wilcoxon signed-rank test. A *P*-value of < 0.05 was considered significant.

## Results

### Changes in clinical outcomes

Comparing the preoperative values with the 2-year postoperative values, the average UCLA score increased from 9.4 ± 4.4 to 29.7 ± 3.9 in group A and from 9.8 ± 4.0 to 29.3 ± 3.6 in group B. The JOA score increased from 52.5 ± 6.4 to 87.4 ± 6.1 in group A and from 50.1 ± 7.5 to 87.4 ± 8.6 in group B (Table 2). The average range of forward elevation increased from 75.0 ± 9.8 to 129.1 ± 20.1 degrees in group A and from 74.4 ± 20.1 to 133.9 ± 24.1 degrees in group B. External rotation increased from 17.9 ± 10.4 to 29.1 ± 12.7 degrees in group A and from 22.9 ± 14.9 to 29.4 ± 10.7 in group B. Internal rotation increased from 1.8 ± 0.9 to 2.4 ± 1.0 points in group A and from 1.9 ± 0.8 to 2.3 ± 1.1 points in group B. The average NRS score improved from 8.2 ± 0.9 to 0.9 ± 1.2 in group A and from 7.9 ± 0.7 to 1.1 ± 1.3 in group B. Both groups showed significant postoperative improvement in UCLA scores, JOA scores, ROM, and NRS scores (*P* < 0.001). In both groups, no significant differences were found between preoperative and 2-year postoperative follow-up values or in the changes that occurred preoperatively and 2 years postoperatively (Table 3).

**Table 2:** Comparison of the clinical outcome between group A and group B

	Preoperatively		2 years postoperatively		*P*-value
	Mean ± SD	*P*-value	Mean ± SD	*P*-value	
UCLA score					
Group A	9.4 ± 4.4	0.723	29.7 ± 3.9	0.668	< 0.001*
Group B	9.8 ± 4.0		29.3 ± 3.6		< 0.001*
JOA score					
Group A	52.5 ± 6.4	0.168	87.4 ± 6.1	0.439	< 0.001*
Group B	50.1 ± 7.5		87.4 ± 8.6		< 0.001*

Group A: RSA for failed ARCR, Group B: Primary RSA

*SD* standard deviation, *JOA* Japanese Orthopaedic Association, *UCLA* The University of California, Los Angeles

*There was a significant difference

**Table 3:** Comparison of ROM and pain scale between group A and group B

	Preoperatively		2 years postoperatively		*P*-value
	Mean ± SD	*P*-value	Mean ± SD	*P*-value	
Anterior elevation (degrees)					
Group A	75.0 ± 9.8	0.608	129.1 ± 20.1	0.114	< 0.001*
Group B	74.4 ± 20.1		133.9 ± 24.1		< 0.001*
External rotation (degrees)					
Group A	17.9 ± 10.4	0.229	29.1 ± 12.7	0.546	0.011*
Group B	22.9 ± 14.9		29.4 ± 10.7		< 0.001*
Internal rotation (points)					
Group A	1.8 ± 0.9	0.346	2.4 ± 1.0	0.389	< 0.001*
Group B	1.9 ± 0.8		2.3 ± 1.1		< 0.001*
NRS					
Group A	8.2 ± 0.9	0.549	0.9 ± 1.2	0.949	< 0.001*
Group B	7.9 ± 0.7		1.1 ± 1.3		< 0.001*

Group A: RSA for failed ARCR, Group B: Primary RSA

*SD* standard deviation, *JOA* Japanese Orthopaedic Association, *UCLA* University of California, Los Angeles

*There was a significant difference

### Presence of complications

One (4.0%) and two (3.3%) complications were observed in groups A and B, respectively, without a significant difference between the groups. In group A, the complication included a postoperative dislocation, which was treated with closed reduction. In group B, complications included a postoperative dislocation, which required reoperation for partial prosthesis exchange, and a postoperative acromion fracture, which was treated conservatively (Table 4).

**Table 4:** Comparison of the complication rate between group A and group B

	Group A (*n* = 25)	Group B (*n* = 60)	*P*-value
Complication (+)	1	2	0.879
Complication (-)	24	58	
Complication rate	4.0%	3.3%	

Group A: RSA for failed ARCR, Group B: Primary RSA

## Discussion

This study demonstrated that RSA could improve functional outcomes in patients with or without prior ARCR. Our hypotheses were confirmed, as patients with a history of ARCR showed significant improvements, with no difference in outcomes compared to those without any prior surgeries.

RSA is a well-established method in the treatment of CTA, massive rotator cuffs, proximal humeral fractures, and fracture sequelae. The number of RSA procedures performed in Japan has been increasing since its introduction in 2014. ARCR has an established role in treating rotator cuff tears. With the aging population, the number of ARCRs being performed continues to rise. Consequently, both RSA and ARCR are increasingly being performed at our institution. Despite ARCR being a well-established method, there are many reports of an approximate retear rate of 15–32% [17–21], which remains a significant concern. The optimal treatment of failed ARCR remains a topic of discussion and, thus, is an important issue that requires further clarification. Many reports have discussed options for patients experiencing a retear after ARCR, including arthroscopic debridement, re-repair, and superior capsular reconstruction [22–26]. We believe that it is difficult to decide the method of operation, especially if the tear is irreparable. Among these alternatives, RSA has emerged as an effective treatment for managing failed ARCR. The rising incidence of reverse shoulder arthroplasty procedures partially reflects increasing revision cases following failed primary rotator cuff repairs.

Previous studies have identified a prior surgery as a negative predictor for postoperative function in patients undergoing RSA. Inferior outcomes have been reported when RSA is performed as a revision procedure, particularly following a failed arthroplasty or failed osteosyntheses, compared with primary RSA [4, 19, 20].

Koeppe et al. compared RSA after a failed locked plate fixation and primary RSA in the treatment of proximal humeral fractures. They stated that elderly patients benefit from a one-stop operation because it might prevent complications or revision surgery, and if in doubt, surgeons should decide in favor of primary RSA [15]. However, the impact of prior ARCR on RSA outcomes remains unclear and controversial.

Some studies have reported that prior ARCR does not negatively affect RSA outcomes. Erickson et al. found significant improvements in the American Shoulder and Elbow Surgeons scores following RSA in patients with prior rotator cuff repair, suggesting that previous rotator cuff repair does not significantly impact RSA outcomes [5]. Patel et al. demonstrated similar functional outcomes and complication rates between patients undergoing RSA with and without prior rotator cuff repair [8]. Similarly, Vervaecke et al. reported that RSA after retear of ARCR does not increase costs, readmission rates, or healthcare resource utilization compared with primary RSA [11]. In our previous study comparing RSA and ASCR after ARCR, we found that RSA yielded superior outcomes in ROM, NRS, and clinical scores compared with ASCR and was comparable to primary RSA [12].

In contrast, other studies have reported inferior outcomes in patients with prior rotator cuff repair compared with those undergoing primary RSA. Marigi et al. noted that functional outcomes in patients undergoing RSA after rotator cuff repair were slightly worse than those in patients without prior surgery [7]. Shields et al. found that patients undergoing RSA with prior rotator cuff repair experienced fewer short-term gains than did those without prior shoulder surgery [9]. Additionally, Boileau et al. reported that although RSA improves functional outcomes after failed cuff repair, the results remain inferior to those of primary RSA; therefore, surgeons should inform the patient about the potential loss of motion [13].

Our study found no significant differences in outcomes between patients undergoing RSA with or without prior ARCR. These findings reinforce that RSA provides substantial functional improvements in patients with previous rotator cuff repair and that prior ARCR does not significantly impact ROM, NRS scores, clinical scores, or complication rates following RSA. Therefore, our study supported the opinion that “prior ARCR does not negatively affect RSA outcomes,” reported in the existing literature.

Determining the most appropriate surgical approach remains a challenge in orthopedic practice. Assessing whether a rotator cuff is amenable to primary repair is difficult, and many patients prefer to avoid arthroplasty due to its invasiveness, even when recommended. Erickson et al. noted that many patients initially opt for an attempted repair to delay arthroplasty, which is a reasonable approach since prior rotator cuff repair does not appear to compromise RSA outcomes. Our data supports this perspective, and we believe that these findings can assist in surgical decision-making in challenging cases.

We believe that ARCR can be recommended as the primary approach in such cases because RSA can achieve similar improvements in ROM, NRS, and clinical scores, even with prior ARCR, and that prior ARCR does not compromise the outcomes of a subsequent RSA. For patients who are afraid of or cannot consent to artificial joint surgery, recommending ARCR first may be an option. We expect that even if ARCR fails, we can suggest and perform RSA, which would lead to improvement and satisfaction. Revision surgeries are generally associated with challenges, such as tissue adhesions, increased bleeding, and higher invasiveness, making recovery more difficult. Shields et al. reported that patients with prior ARCR had inferior primary RSA in these results. They added that lower functional scores in patients with prior ARCR might be due to postoperative scarring or psychological factors related to requiring another surgery and another rehabilitation [9]. However, our findings indicate that RSA after ARCR yields similar outcomes to those of primary RSA. ARCR is a less invasive procedure for soft tissue and joints compared with arthroplasty or osteosynthesis. Because of its minimally invasive nature, adhesions and bleeding remain minimal even in subsequent RSA, making the procedure less technically demanding. Moreover, RSA is designed for cases where rotator cuff function is lost, and in RSA following failed ARCR, there is no need to re-repair the torn rotator cuff. This may help mitigate the difficulties typically associated with reoperations. By fundamentally altering joint mechanics, RSA can compensate for poor soft tissue conditions, bleeding from surrounding tissues, and stiffness from prior ARCR, leading to outcomes comparable to those of primary RSA.

Therefore, we believe that RSA after ARCR is less invasive than revision surgery following arthroplasty or osteosynthesis, minimizing the impact of prior surgery. Based on our findings, even if ARCR fails, it is unlikely to significantly affect the outcomes of subsequent RSA. Thus, ARCR may still be considered as the initial treatment, particularly for younger patients or those seeking to avoid the highly invasive nature of arthroplasty, even in cases of large or massive rotator cuff tears.

However, RSA should remain a “last resort,” and its indications must be carefully evaluated. Further research is needed to explore the effects of prior ARCR and other factors influencing RSA outcomes, as well as to assess long-term follow-up results.

Our study had several limitations. The sample size was relatively small, and the follow-up period was short. Additionally, we did not account for factors, such as rotator cuff tear size or the number of anchors used in prior ARCR, which may have influenced our findings. These limitations should be considered when interpreting the results.

## Conclusions

Our results demonstrated the efficacy of RSA in patients with or without prior ARCR. RSA following prior ARCR showed similar clinical outcomes and complication rates to RSA without prior ARCR. The study proved that prior ARCR is not a negative predictor of RSA.

## Data Availability

The study data used to support the findings of this study are available from the author upon request.

## Abbreviations

RSA: Reverse shoulder arthroplasty
CTA: Cuff tear arthropathy
ARCR: Arthroscopic rotator cuff repair
MRI: Magnetic resonance imaging
ROM: Range of motion
RA: Rheumatoid arthritis
UCLA: The University of California, Los Angeles
JOA: Japanese Orthopaedic Association
NRS: Numerical Rating Scale
